# Astronomical

**Published:** 1867-07

**Authors:** 


### *Astronomical.*

Our friend, Dr. H., overheard a *nymph du pave* request a good-looking stranger to retire awhile and spend "a few moments with Venus." "I beg pardon," was the prompt reply. "The last time I spent a few moments with Venus I was obliged afterwards to spend six months with Mercury."

